# E-Waste and Harm to Vulnerable Populations: A Growing Global Problem

**DOI:** 10.1289/ehp.1509699

**Published:** 2015-09-29

**Authors:** Michelle Heacock, Carol Bain Kelly, Kwadwo Ansong Asante, Linda S. Birnbaum, Åke Lennart Bergman, Marie-Noel Bruné, Irena Buka, David O. Carpenter, Aimin Chen, Xia Huo, Mostafa Kamel, Philip J. Landrigan, Federico Magalini, Fernando Diaz-Barriga, Maria Neira, Magdy Omar, Antonio Pascale, Mathuros Ruchirawat, Leith Sly, Peter D. Sly, Martin Van den Berg, William A. Suk

**Affiliations:** 1 National Institute of Environmental Health Sciences, National Institutes of Health, Department of Health and Human Services, Research Triangle Park, North Carolina, USA; 2 MDB, Inc., Durham, North Carolina, USA; 3 Water Research Institute, Council for Scientific and Industrial Research, Accra, Ghana; 4 Swedish Toxicology Sciences Research Center, Södertälje University, Södertälje, Sweden; 5 World Health Organization, Geneva, Switzerland; 6 University of Alberta, Edmonton, Alberta, Canada; 7 University at Albany, Rensselaer, New York, USA; 8 University of Cincinnati, Cincinnati, Ohio, USA; 9 Shantou University Medical College, Shantou, China; 10 Basel Convention Regional Centre for Training and Technology Transfer, Al-Orman, Giza, Egypt; 11 Icahn School of Medicine at Mount Sinai, New York, New York, USA; 12 United Nations University Institute for Sustainability and Peace, United Nations University, Bonn, Germany; 13 Universidad Autónoma de San Luis Potosí, San Luis Potosí, Mexico; 14 Cairo University, Giza, Egypt; 15 Department of Toxicology, School of Medicine, University of the Republic, Montevideo, Uruguay; 16 Chulabhorn Research Institute, Bangkok, Thailand; 17 Queensland Children’s Medical Research Institute, Brisbane, Queensland, Australia; 18 Utrecht University, Utrecht, the Netherlands

## Abstract

**Background:**

Electronic waste (e-waste) is produced in staggering quantities, estimated globally to be 41.8 million tonnes in 2014. Informal e-waste recycling is a source of much-needed income in many low- to middle-income countries. However, its handling and disposal in underdeveloped countries is often unsafe and leads to contaminated environments. Rudimentary and uncontrolled processing methods often result in substantial harmful chemical exposures among vulnerable populations, including women and children. E-waste hazards have not yet received the attention they deserve in research and public health agendas.

**Objectives:**

We provide an overview of the scale and health risks. We review international efforts concerned with environmental hazards, especially affecting children, as a preface to presenting next steps in addressing health issues stemming from the global e-waste problem.

**Discussion:**

The e-waste problem has been building for decades. Increased observation of adverse health effects from e-waste sites calls for protecting human health and the environment from e-waste contamination. Even if e-waste exposure intervention and prevention efforts are implemented, legacy contamination will remain, necessitating increased awareness of e-waste as a major environmental health threat.

**Conclusion:**

Global, national, and local levels efforts must aim to create safe recycling operations that consider broad security issues for people who rely on e-waste processing for survival. Paramount to these efforts is reducing pregnant women and children’s e-waste exposures to mitigate harmful health effects. With human environmental health in mind, novel dismantling methods and remediation technologies and intervention practices are needed to protect communities.

**Citation:**

Heacock M, Kelly CB, Asante KA, Birnbaum LS, Bergman AL, Bruné MN, Buka I, Carpenter DO, Chen A, Huo X, Kamel M, Landrigan PJ, Magalini F, Diaz-Barriga F, Neira M, Omar M, Pascale A, Ruchirawat M, Sly L, Sly PD, Van den Berg M, Suk WA. 2016. E-waste and harm to vulnerable populations: a growing global problem. Environ Health Perspect 124:550–555; http://dx.doi.org/10.1289/ehp.1509699

## Introduction

Electronic product innovations satisfy many needs, including the desire of people to stay connected around the globe. As new products are continually introduced into the marketplace, consumers replace existing electronic products that are damaged or simply outdated. The resulting mass of electronic products discarded is becoming the fastest-growing waste stream in the world (Lundgren 2012), leading to polluted environments. E-waste refers to all types of electrical or electronic equipment (EEE) and its parts that have been discarded without intention for reuse by the owner [StEP (Solving the E-Waste Problem) Initiative 2014a]. Global e-waste generation was estimated to be 41.8 million tonnes in 2014 and may increase to 65.4 million tonnes by 2017 (Breivik et al. 2014).

E-waste is globally recognized as a resource because of the potential for recovering valuable materials including iron, aluminum, copper, gold, silver, and rare earth metals. However, electronic products have not been designed to efficiently recover these valuable materials or for their safe disposal. Only an estimated 15% of global e-waste is fully recycled (Modak 2011) to extract these valuable materials even though economic benefits can result from their recovery.

In many low- and middle-income countries, handling and disposal of discarded EEE is frequently unregulated. Safety concerns arise because e-waste also contains hazardous constituents such as lead, mercury, and chromium, certain chemicals in plastics, and flame retardants. Documentation is increasing about health effects related to contamination in air, soil, and water for people working and living at or near informal e-waste processing sites (Grant et al. 2013). Evidence of adverse health effects and the increasing number of e-waste sites make protecting human health and the environment from e-waste contamination an expanding challenge.

This commentary is the result of discussion and subsequent consensus among members of a sub-workgroup that was part of an international working group meeting on e-waste and children’s health, convened in June 2013 at the World Health Organization (WHO) Headquarters in Geneva, Switzerland. Composed of diverse experts, the workgroup declared a need for and discussed strategies to reduce exposure to the harmful elements in e-waste (Alabaster et al. 2013).

Here, before describing adverse health effects from e-waste exposures, we provide context through a brief overview of the scale and risks associated with this rapidly increasing hazardous waste stream. We next review international efforts concerned with environmental hazards, especially those affecting children. Building from recommendations from the 2013 WHO workgroup, we recommend the need for international cooperation to raise awareness about e-waste as a major environmental health threat (Alabaster et al. 2013). We conclude by addressing next steps to devise and build upon solutions for intervention or prevention of harm from informal e-waste processing.

## Discussion

*E-waste scale and flow.* Massive amounts of e-waste are produced globally and redistributed. According to 2014 estimates, the top producer was the United States, which generated 7.1 million tonnes (Mt), followed by China, which generated nearly 6.0 Mt (Baldé et al. 2015). When the amount of e-waste produced is considered per person, the countries within Europe generated an average of 15.6 kg of e-waste per inhabitant (Baldé et al. 2015). China and certain countries within Africa receive up to 80% of the world’s e-waste (Lundgren 2012).

Although e-waste is not generated exclusively by wealthy countries, such countries contribute substantially to e-waste problems in low- to middle-income countries because of regulatory ambiguities that allow EEE export for re-use, regardless of actual product functionality. Consequently, the export of much discarded EEE infringes transboundary shipment frameworks, such as the Basel Convention, that are meant to reduce waste shipment across national or other political borders [United Nations Environment Programme (UNEP) 2011]. Consequently, e-waste is shipped to countries that often lack adequate infrastructure to effectively manage it in an environmentally sound manner (Modak 2011). For example, Ghana has an unregulated and unrestricted import regime for secondhand EEE. In 2009, 70% of EEE imports into Ghana were designated as secondhand products, but considerable amounts of these imports were near or at end of life and quickly designated as waste because they had little or no utility value (Amoyaw-Osei et al. 2011).

The Agbogbloshie area of Ghana, where about 40,000 people live, provides an example of how e-waste contamination can pervade the daily lives of nearly all residents. Into this area—one of the largest informal e-waste dumping and processing sites in Africa—about 215,000 tons of secondhand consumer electronics, primarily from Western Europe, are imported annually. Because this region has considerable overlap among industrial, commercial, and residential zones, Pure Earth (formerly Blacksmith Institute) has ranked Agbogbloshie as one of the world’s 10 worst toxic threats (Blacksmith Institute 2013).

*Chemicals in e-waste and potential for valuable material recovery.* About 60 chemical elements can be found in various complex electronics, including lead, cadmium, chromium, mercury, copper, manganese, nickel, arsenic, zinc, iron, and aluminum, many of which are potentially, or known to be, hazardous (Grant et al. 2013). These metals are used in products such as circuit boards, semiconductor chips, cathode ray tubes, coatings, and batteries. Electronic goods also contain a range of other potentially hazardous chemicals that are part of the manufacturing process, including persistent organic compounds used as fire retardants or found in product fluids, lubricants, and coolants.

Electronics can also contain constituents that are important for recovery. A mobile phone, for example, can contain > 40 elements, including base metals such as copper, special metals such as cobalt, and precious metals such as gold, that are desirable for recycling (StEP Initiative 2014b). Precious and special metals such as platinum, indium, and ruthenium that are used extensively in modern electronics are naturally available in limited amounts (Schluep et al. 2009). Although the amount of material, such as copper, needed for any one mobile phone is minuscule, when 6 billion global mobile-cellular subscriptions are considered (International Telecommunications Union 2012), it becomes clear that electronic products are a major driver for the demand of certain metals. This demand is unlikely to subside.

Recycling metals contained in electronic goods may reduce the need for mining virgin materials. However, many of these valuable resources are lost every day through low e-waste collection rates and inadequate recycling or low-efficiency end processing for EEE (StEP Initiative 2011). It is estimated that only 25% of valuable metals are recovered during informal e-waste recycling (UNEP 2008). Although an efficient, formal recycling sector exists in some developed countries, this sector does not provide feasible solutions for unregulated, informal e-waste processing. Proper and efficient recycling, which recovers valuable materials with minimal environmental harm, is intricate and expensive (Huisman et al. 2007).

*Economic considerations.* Although the e-waste stream is a small portion of global municipal waste, accounting for about 5% (Modak 2011), it plays a significant employment role in the recycling sectors of some low- and middle-income countries (Modak 2011) such as China, India, Pakistan, Malaysia, Thailand, the Philippines, Vietnam, Ghana, and Nigeria (Lundgren 2012). For example, in Guiyu, China, possibly the largest e-waste recycling location in the world, about 100,000 people are employed as e-waste recyclers (Lundgren 2012).

Ideally, the collection of electrical and electronic products is a sustainable process that maximizes recycling to retain valuable e-waste components in the economy and safely disposes of dangerous components (Schluep et al. 2009). Efforts are underway to move toward a more sustainable process such as better control of the transboundary movements of e-waste. Additionally, the StEP Initiative, a coordinated global effort, brings expertise to meet the social, political, economic, and environmental challenges of extracting valuable resources from e-waste. Some low- and middle-income countries, including Nigeria and Egypt, are working toward increased regulation or accountability for used EEE (Chaplin and Westervelt 2015). For instance, in Egypt the importation of working EEE > 5 years old is banned (Chaplin and Westervelt 2015). Even after corrective actions are implemented for legal EEE movement, sustainable product design, and proper recycling practices, legacy environmental problems from amassed e-waste will remain. Moreover, in the interim, basic economics drives the e-waste business by fostering maximization of profits without safe treatment and disposal of hazardous parts.

*Labor practice considerations.* The unregulated and unaccountable collecting, processing, and redistributing of unwanted EEE tends to be performed by workers at temporary sites, residences, crude workshops, and open public spaces. Communities with primitive, informal recycling operations tend to be populated by poor people with scarce job possibilities who are desperate to feed themselves and their families, and this primary concern overrides that for personal health and safety (Lancet 2013). These workers may use risky processing practices without knowledge of or access to exposure-minimizing technology or personal protective equipment (Lancet 2013). And, alarmingly, children are commonly employed in e-waste recycling because their small hands make them ideal to dismantle equipment (Lancet 2013).

Common primitive labor practices include using acid baths, burning cables, breaking apart toxic solders, and dumping consequent waste material (StEP Initiative 2011). When acid baths are used to extract precious metals such as gold, a lack of protective clothing leaves recyclers at high risk of chemical injury. Workers dismantling e-waste may come into direct contact with polychlorinated biphenyls (PCBs) and other persistent organic pollutants in fluids, lubricants, and coolants (Grant et al. 2013). Processing cables to recover valuable copper often involves burning the plastic coating from wires, which releases harmful polyvinyl chloride, dioxins, furans, brominated flame retardants, and polycyclic aromatic hydrocarbons (PAHs) into the environment. While the cables burn, the immediate environment where people work and live is engulfed in thick black toxic smoke (Asante et al. 2012). The harmful combustion byproducts released while burning e-waste can increase risk of respiratory and skin diseases, eye infections, and even cancer for people nearby (Robinson 2009). As a consequence, workers and people playing or living in or near informal facilities are chronically exposed to myriad chemical pollutants either directly through contact or inhalation, or indirectly through contamination of the food and water supply (Lancet 2013).

*E-waste exposure and health risks.* E-waste exposure is a complex process because there are many routes and sources, different exposure time periods, and possible inhibitory, synergistic, or additive effects of chemicals (Grant et al. 2013). Exposure variability may come from the type and quantity of e-waste, length of processing history at sites, and methods and locations of processing activities and physiological vulnerability, especially in pregnant women and children. Although the extent to which contamination from e-waste contributes to adverse health effects is not known, it is believed to be a significant factor in or near communities where informal recycling takes place. Studies conducted in China and India indicate that hazardous substances from e-waste can extend beyond processing sites and into ecosystems (Sepúlveda et al 2010; Zhang et al. 2011).

People are exposed to hazardous substances in e-waste through multiple routes, including water, air, soil, dust, and food. (Norman et al. 2013). Cumulative exposures are predictably high where informal recycling sites have operated for more than a decade (Chen et al. 2011). For example, rice and dust samples collected from homes close to e-waste sites had concentrations of lead, cadmium, and copper that were nearly twice the maximum permissible concentrations (Zheng et al. 2013). An exposure of contaminated food such as rice combined with inhaling lead through house dust puts children at high risk for neurotoxicity and adverse developmental effects (Zheng et al. 2013).

Because of the unique ways in which children interact with the environment, they are likely to receive bigger doses of toxicants, relative to their size, than adults. Diet is an important exposure source, and children eat more food and drink more water per pound of body weight than do adults (Suk et al. 2003). Breast milk from mothers at e-waste sites indicates elevated exposure to toxicants, such as dioxin, compared with milk from mothers at a reference site (Asante et al. 2011; Chan et al. 2007). Frequent hand-to-mouth behavior in younger children can increase exposure to chemicals from dust or play items (Landrigan et al. 1998). Chemicals can accumulate in children’s bodies because their immature systems are unable to process and excrete some toxic materials effectually (Suk et al. 2003).

Whether the exposure is direct or indirect, the health and environmental effects from many of the individual hazardous substances often found in e-waste are well established from existing studies, including studies in children (Grant et al. 2013; Lundgren 2012). A published review of e-waste and child health included residential and occupational exposures, specific chemical and physical hazards, recent research advances, and methodologies used in exposure assessment (Grant et al. 2013). Studies included in the review confirmed that serum in children and pregnant women contained many contaminants found in e-waste. Grant et al. (2013) concluded that health consequences from e-waste exposure are plausible and may include changes in thyroid function, altered cellular expression and function, adverse neonatal outcomes, cognitive and behavioral changes, and decreased lung function.

Several known developmental neurotoxicants are found in e-waste, such as lead, mercury, cadmium, and brominated flame retardants, which can lead to irreversible cognitive deficits in children and behavioral and motor skill dysfunction across the lifespan (Chen et al. 2011). Children may directly encounter hazardous substances in fumes or dust through inhalation, skin contact, or oral intake via dismantling activities they perform themselves or that are performed by others nearby (Grant et al. 2013). Indirect exposure routes for children, as well as for highly susceptible fetuses, also involve polluted air and drinking water (Grant et al. 2013). Exposure variability among children also depends on parental involvement at processing sites, either in or away from the home, and the daily activities of the child (Chen et al. 2011).

In summary, the health of many people, with particular concern for children, is harmed by the contamination resulting from e-waste. Hazardous substances move from discarded EEE across the environment where people are exposed through air, water, soil, and even the food they eat. Thus the threat of adverse environmental health is immediate in many places that accept and informally handle e-waste.

*International coordination and collaboration efforts.* The e-waste problem has been building for decades. The transboundary shipment and disposal of hazardous wastes attracted attention in the 1980s when some industrialized countries indiscriminately sought less expensive disposal of their hazardous wastes abroad (Cunningham and Cunningham 2004; Vir 1989), resulting in the Basel Convention (UNEP 2011). Subsequently, environmental threats to susceptible populations were considered by international groups over the years. Although not all efforts described here specifically addressed e-waste, in total the activities show progress toward addressing current e-waste problems. Briefly described in chronological order, this compilation of international efforts leads to our suggested next steps to build on existing efforts to reduce or prevent harm from e-waste exposures.

Basel Convention. Negotiated under the auspices of the United Nations Environment Programme and entered into force in 1992, the convention regulates the transboundary movement and disposal of hazardous and other wastes. Its overarching objective is to protect human health and the environment against the adverse effects of hazardous wastes (UNEP 2011). Under the Basel terms, based on the concept of prior informed consent, an export may proceed only with written consent by the country of import. However, the terms are difficult to monitor because reliable data are not available regarding the amount of exported EEE that is accurately classified as e-waste.

Bangkok Statement on Children’s Environmental Health. In 2002, in Bangkok, Thailand, an international group of professionals discussed children’s health and environmental threats. They committed to developing networks and urged the WHO to support protection and prevention, health care and research, empowerment and education, and advocacy to translate knowledge into action for improving the environmental health of children (WHO 2002).

Solving the E-waste Problem Initiative. Officially launched in 2007, the StEP Initiative strives to lead global management and development of environmentally, economically, and ethically sound e-waste recovery, re-use, and prevention. It facilitates research, analysis, and dialogue among representatives from industry, international organizations, governments, nongovernmental organizations, and academic institutions. (StEP Initiative 2011).

Bali Declaration. Adopted in 2008, the Bali Declaration on Waste Management for Human Health and Livelihood affirmed that poorly managed waste may have serious consequences for the environment, human health, and sustainable livelihood. It called for strengthened political cooperation to increase capacity building and to promote and enhance public and private investment for safe and environmentally careful waste management technology (UNEP 2008).

Busan Pledge for Action on Children’s Environmental Health. The third International Conference on Children’s Health and Environment, held in Busan, Republic of Korea, in 2009, acknowledged that old and new environmental threats existed in homes, schools, playgrounds, health care, and other common childhood settings. The resultant pledge urged the WHO to promote the recognition, assessment, and study of environmental factors affecting the health and development of children, specifically including electronic waste (WHO 2009).

Geneva Meeting on E-waste and Children’s Health. Convened by the WHO in 2013, attendees reviewed e-waste and children’s issues including exposures, health concerns, research gaps, and successful interventions (Alabaster et al. 2013). Attendees agreed to raise awareness of the threat of e-waste to children’s health; to establish a network of experts and international stakeholders concerned with e-waste management; and to plan, prepare, and engage in concrete interventions to prevent and reduce exposures. They issued the Geneva Declaration on E-waste and Children’s Health, which states that scientific information is sufficient to support a concern about the improper management of e-waste (Alabaster et al. 2013).

They also advocated creating ways to ensure the safety of people involved in all lifecycle stages of EEE, with a focus on protecting vulnerable populations, particularly children and maturing embryos and fetuses (Alabaster et al. 2013). The Declaration strongly encourages worldwide implementation of measures to prevent and reduce harmful e-waste exposures and to enforce existing regulations while methodically developing more exacting regulations for sound e-waste management. Despite certain data gaps, the authors agreed that the high risk for harm to health justified immediate intervention. They decided that financial, technical, and human resources should be identified and provided to effectively manage public health issues associated with e-waste. They recognized that reducing poverty would, at the same time, alleviate the e-waste challenge in the long-term (Alabaster et al. 2013).

Pacific Basin Consortium for Environment and Health. In response to the Geneva Declaration on E-waste and Children’s Health, the 2013 meeting of the Pacific Basin Consortium hosted the official launch of the WHO’s E-waste and Child Health Initiative (Sly 2014; WHO 2013). With a special emphasis on children, the initiative includes recognition of the need to identify the main sources of e-waste exposure and the resulting health effects. At global, national, and local levels, the initiative also seeks to identify prevention and intervention strategies that take into account the socioeconomic status of those who are most vulnerable to e-waste hazards.

*Addressing e-waste problems.* In light of international scrutiny about serious concerns associated with e-waste and environmental health, particularly among vulnerable populations, we considered existing efforts to identify, investigate, and improve conditions associated with e-waste processing. Efforts are underway to clarify terminology used in the Basel Convention to distinguish waste from non-waste (UNEP 2015), and projects such as ZeroWIN (Towards Zero Waste in Industrial Networks) work toward e-waste reduction by addressing the design phase of the electronic life cycle (Luepschen et al. 2013). However, even if these initiatives are successful, legacy contamination of soil, sediment, and water will remain. Intense study is needed to learn how to best remediate this contamination and to understand the effects of resulting exposures. In addition, because of the need for economic survival, realistically, some informal recycling will still occur. Education about potential health effects will be needed for workers as well as people who simply live near previous or current recycling sites. In addition, novel dismantling methods and technologies, along with training on their proper use, are needed to protect and engage communities.

We believe that any framework for e-waste interventions should include the concept of human security. As defined by the United Nations, human security includes seven main categories: economic, food, health, environmental, personal, community, and political (United Nations Development Programme 1994). The goal of human security is to ensure the survival, livelihood, and dignity of people in response to current and emerging threats that are widespread and cross-cutting. E-waste is such a threat. We recommend interventions at the global, national, and local levels with a simple overarching purpose: to achieve safe e-waste recycling operations that recognize and take into account security issues for impoverished people.

In the following sections, we present areas for next steps in addressing the global e-waste problem.

Reduce children’s e-waste exposures to mitigate harmful health effects. Focusing on the health effects of children’s direct or indirect exposure to e-waste must become a priority of the international community (Grant et al. 2013). Children have special periods in their development when they are more susceptible than adults to the effects of many chemical, biological, and physical agents (Suk et al. 2003; WHO 2006). Because we know that many of the individual e-waste constituents are well studied and known to be extremely harmful, we can assume that they are just as harmful, or even more so, in combination.

Children’s special vulnerability and increased susceptibility to adverse health outcomes can be exacerbated by many factors, such as malnutrition (Suk et al. 2003), and will likely require a comprehensive, preventive approach to protection from e-waste harm. These efforts must also include the broad approach to promoting children’s environmental health outlined more than a decade ago in the Bangkok Statement (Suk et al. 2003), which called for addressing human population growth, land and energy use patterns, habitat destruction, biodiversity loss, and climate change. This all-embracing strategy is premised on the concept that intervention can be effective before full attainment of sufficient evidence of causality (Sly et al. 2014). Consideration of the possibility of harm entails following the Precautionary Principle (Suk and Olden 2004), an approach also recommended by the European Ramazzini Foundation of Oncology and Environmental Sciences (Grandjean et al. 2004) because of the potential for exposure to harmful chemicals present in high quantities in e-waste recycling communities (Lancet 2013).

Address protection of e-waste workers. Prevention and intervention strategies related to e-waste contamination must recognize that impoverished people will continue, at least in the short term, to work informally with e-waste recycling. The economic gain derived from the great demand for raw materials such as copper is too powerful to expect otherwise.

As a start toward identifying intervention solutions, a framework from the StEP Initiative, called the “Best-of-2-Worlds,” provides a philosophy and pragmatic approach for e-waste treatment in emerging economies (Wang et al. 2012). In essence, the approach retains manual dismantling of e-waste in low- to middle-income countries, with critical fractions then sent to high-tech end-processing facilities in a global market. Pilot projects in China and India indicated successful implementation of this scheme with environmental, safety, and economic benefits (Wang et al. 2012).

The international community must recognize the economic opportunities that the e-waste sector can yield, such as sustainable recycling that reclaims valuable materials such as copper and rare earth metals. With this understanding in place, policy makers must rise to the challenge of creating safe systems that also educate and protect workers. Multi-sectoral e-waste policies should address environmental, economic, social, and health aspects with a diversity of stakeholders participating in their development and implementation. Developing effective policies could be especially difficult in countries without a solid history of regulating the environment or supporting commerce. Any effort to connect the formal and informal e-waste processing sectors will be a complex task. Additionally, stakeholders concerned with policy development may not be aware of the effectiveness of existing policies (Kuehr and Magalini 2013).

Build a comprehensive knowledge base for interventions. Although the evidence base to support the need for prevention is strong, good intervention and prevention practices are not well developed. As a first step, there is a need to review existing interventions and their effectiveness in protecting the health of e-waste workers and their families. Because every location where e-waste recycling could be addressed will be different, based on local culture and recycling practices, so should be the technical assistance. Innovation should be relative to the local setting. There is a need to develop a robust and inclusive knowledge system about best practices for technical assistance from which localities can select and tailor approaches suitable for their situations.

There is also a need to develop support systems that would facilitate the use of technical assistance services. Inconsistent or deficient technologies, labor skills, labor standards, and financing are hurdles for achieving innovative and sustainable e-waste recycling enterprise. For example, informal recyclers often focus on the fastest and least expensive extraction of the most valuable components of e-waste, and protective processes or equipment that reduce profit or increase processing time tend to be viewed as undesirable (Kuehr and Magalini 2013). These workers will require occupational health guidance, tools, and prevention measures that raise awareness about toxicant exposures and provide protection for themselves and children. Embracing these changes is desirable from an economic as well as a health perspective.

Engineer solutions that protect and engage communities. E-waste operations should implement protective and exposure-reduction efforts for workers and their entire communities. Additionally, the long-term disposal of hazardous parts and equipment that cannot be reused must be addressed to avoid legacy contamination and to protect communities. Solving the e-waste problem may also entail fostering EEE redesigns to lengthen product life cycles.

Improving environmental conditions where local concerns and needs are actively considered can be achieved through community-informed engineering solutions. A model that considers local conditions for research and prevention, intervention, and remediation strategies to reduce exposure to e-waste may be found in the U.S. National Institutes of Health, National Institute of Environmental Health Sciences (NIEHS), Superfund Basic Research and Training Program (SRP) (Suk et al. 1999). In the SRP, researchers use a model to find solutions where engineers work directly with public health professionals in communities to devise solutions that make local environments, and the people who live in them, healthier. This model provides an effective interface between researchers and governmental agencies and helps coordinate related activities. It also facilitates translation of essential information to make it understandable among different professional disciplines, which is important for effective communications and practices within affected communities. This multi-directional engagement helps develop a broader understanding necessary to address relevant environmental health issues.

International researchers, public health practitioners, and policy makers have touched on ways to employ actionable solutions. The WHO (2013), working with an international group of nearly 20 people, developed a training package for health care providers to improve diagnosis, prevention, and management of childhood diseases linked to environmental exposures. Human-centered design is another engagement approach that can be used to create strategies that meet people’s desire for change and produce solutions that are technically and organizationally feasible as well as financially viable. These and other methods could be adopted by organizations investigating and addressing e-waste problems.

## Conclusion

Through international cooperation, we can raise awareness about the potential harm that e-waste processing poses to children’s health. We seek to increase the awareness of the environmental health issues triggered by e-waste, which profoundly affect the poor, and to encourage multi-pronged interventions. As a start, the environmental threat to children’s health triggered by e-waste is a focus of a WHO environmental health collaborating center at NIEHS (2014). Through this center, the network formed at the Geneva Meeting on E-waste and Children’s Health (Alabaster et al. 2013) will be strengthened to address prevention and intervention strategies. We would like to see our suggestions further explored through other collaborative avenues as well.

Separately, the WHO will play an important role in identifying interventions, raising awareness of health risks, and in communicating with international policy makers to address e-waste challenges. The NIEHS and its connections to university-based scientists and small-business researchers can help provide the framework for trans-disciplinary investigations into children’s exposures to hazardous substances from e-waste. Additionally, schemes to increase capacity building, reduce exposures, and prevent or decrease the burden of diseases among children due to e-waste should be addressed whenever international stakeholders convene around children’s environmental health.
